# Evaluation of mediastinal lymph nodes using ^18^F-FDG PET-CT scan and its histopathologic correlation

**DOI:** 10.4103/1817-1737.74270

**Published:** 2011

**Authors:** Arvind Kumar, Roman Dutta, Umashankkar Kannan, Rakesh Kumar, Gopi Chand Khilnani, Siddhartha Datta Gupta

**Affiliations:** *Department of Surgical Disciplines, New Delhi, India*; 1 *Nuclear Medicine, All India Institute of Medical Sciences, New Delhi, India*; 2 *Medicine, All India Institute of Medical Sciences, New Delhi, India*; 3 *Department of Pathology, All India Institute of Medical Sciences, New Delhi, India*

**Keywords:** FDG PET-CT, lymphoma, mediastinal lymph nodes, tuberculosis, sarcoidosis

## Abstract

**AIMS AND OBJECTIVES:**

To determine the efficacy of integrated ^18^F-fluorodeoxy glucose positron emission tomography-computed tomography (^18^F-FDG PET-CT) in the evaluation and characterization of mediastinal lymph nodes into benign and malignant pathology.

**METHODS:**

Thirty-five patients with mediastinal lymphadenopathies without primary neoplastic or infective lung pathologies were included in the study. The lymph nodes were detected on contrast-enhanced CT scan of the chest. All patients underwent ^18^F-FDG PET-CT scan for evaluation of mediastinal lymph nodes. Results of PET-CT were compared with histopathology of the lymph nodes and sensitivity, specificity, positive predictive value, negative predictive value, and accuracy were calculated.

**STATISTICAL ANALYSIS:**

The data were collected prospectively and analyzed using (SPSS Inc., Chicago, IL) 11.5 software.

**RESULTS:**

Histopathology results in 35 patients revealed tuberculosis in 12, sarcoidosis in 8, and lymphoma in 15. Maximum standardized uptake value (SUVmax) of the benign lymph nodes ranged from 2.3 to 11.8 with a mean±standard deviation (SD) of 5.02±3.26. SUVmax of the malignant lymph nodes ranged from 2.4 to 34 with a mean±SD of 10.8±8.12. There was a statistically significant difference between benign and malignant pathology (*P*<0.0059). ^18^F-FDG PET-CT has sensitivity of 93% and specificity of 40% with SUVmax 2.5 as the cutoff. We found the optimal SUVmax cutoff to be 6.2 as determined by the receiver–operator characteristic curve. With 6.2 as cutoff, the sensitivity, specificity, and accuracy were 87%, 70%, and 77%, respectively.

**CONCLUSION:**

In countries where tuberculosis and other granulomatous diseases are endemic, SUVmax cutoff value of 2.5 has low specificity. Increasing the cutoff value can improve the specificity, while maintaining an acceptable sensitivity.

Enlarged mediastinal lymph nodes is a common clinical condition encountered by Chest physicians. In the majority of clinical situations, we encounter multiple lymph node enlargements but rarely isolated lymphadenopathy is also encountered. Lymph nodes may be enlarged due to a variety of inflammatory, infectious, or malignant reasons. Hence, it is important to establish a diagnosis and differentiate benign from malignant lymph nodes. Computed tomography (CT) and magnetic resonance imaging (MRI) have been the standard imaging tools for evaluation of enlarged mediastinal lymph nodes, which have their own limitations. CT has low sensitivity (64%) and specificity (62%) in detecting malignant lymph nodes and it makes formal lymph node sampling by mediastinoscopy, thoracoscopy, or thoracotomy necessary to detect metastases.[1] MRI can overlook calcification in lymph nodes and label such enlarged lymph nodes as metastatic deposits. MRI has poor spatial resolution and as a result of that, a group of discrete, adjacent, normal-sized nodes appears as a single large nodal mass, which may be erroneously misdiagnosed as metastatic disease.[2] Although CT and MRI provide good structural details, their diagnostic criteria of size of 1 cm or more for abnormality can overlook microscopic metastases or partial infiltration of the node. ^18^F-Fluorodeoxy glucose positron emission tomography (^18^F-FDG-PET) can detect physiologic and biochemical processes in the body noninvasively. FDG-PET has high sensitivity (84%) and specificity (89%) in detecting malignant pathology in mediastinal lymph nodes in patients of lung cancer.[3] However, FDG-PET alone has limitation of poor localization of abnormal FDG metabolism. It has been reported that CT or PET alone may provide misdiagnosis in differentiating benign from malignant lymph nodes in a substantial proportion of patients. Combined PET-CT has been found to overcome limitations of CT or PET alone as it provides structural and functional information of disease status in the samesetting.[4] Most of the published studies from developed countries have shown high sensitivity and specificity when maximum standardized uptake value (SUVmax) of 2.5 was used as the cutoff value to differentiate benign from malignant conditions. However, these high diagnostic values of PET and PET-CT cannot be extrapolated to countries where mediastinal lymph node involvement due to granulomatous diseases is common. Hence, the present prospective study was planned to explore the role of ^18^F-FDG PET-CT scan in the evaluation of mediastinal lymphadenopathies with no primary neoplastic or infective lung pathology in a population in which infective and inflammatory diseases in the mediastinum are common.

## Methods

This prospective observational study was carried out in a tertiary care referral hospital after approval from the Institutional Ethical Committee. Thirty-five patients with mediastinal lymphadenopathy detected on contrast-enhanced CT chest with no evidence of primary neoplastic or infective lung pathology were included in this study. There were 23 males and 12 females with mean age of 42.74 years. Characteristics of these patients are provided in Table 1. All the patients underwent combined ^18^F-FDG PET-CT scan for evaluation of mediastinal lymphadenopathies. Results of PET-CT were compared with histopathology, which was considered as “gold standard” and sensitivity, specificity, positive predictive value (PPV), negative predictive value (NPV), and accuracy of PET-CT were calculated.

**Table 1:** Clinical, histology, and ^18^F-FDG PET-CT imaging characteristics of the patients

Characteristics	Overall	Sarcoidosis	Tuberculosis	Lymphoma	
				Hodgkin’s lymphoma	Non-Hodgkin’s lymphoma
Number of patients	35	8 (23%)	12 (34%)	10 (29%)	5 (14%)
Mean age (years)	42.74	40.62	40.33	44.80	47.80
Sex (Male/Female)	23/12	4/4	5/7	9/1	5/0
Lymph node size (cm)					
Mean	3.48	2.66	3.02	3.98	4.94
Median	2.80	2.30	2.85	3.00	4.50
SD	2.51	1.08	0.96	3.96	2.90
SUVavg					
Mean	3.29	2.21	2.38	4.84	5.44
SD	3.11	1.36	1.30	4.14	5.76
SUVmax					
Mean	7.53	4.61	5.29	10.52	6.78
SD	6.46	3.20	3.42	5.41	3.70
Biopsy					
PLNB	19	04	05	08	02
TBLB	06	02	04	—	—
MS	03	02	01	—	—
EUSBx	01	—	01	—	—
CTBx	06	—	01	02	03
Total	35	08 (23%)	12 (34%)	10 (29%)	05 (14%)

^18^F-FDG PET-CT, ^18^F-fluorodeoxy glucose positron emission tomography-computed tomography; SD, standard deviation; TBLB, transbronchial lymph node biopsy; MS, mediastinoscopy; PLNB, peripheral lymph node biopsy; EUSBx, endoscopic ultrasound-guided biopsy; CTBX, CT-guided biopsy; SUVmax, maximum standardized uptake value; SUVavg, average standardized uptake value.

### ^18^F-FDG-PET-CT scan protocol

All the patients were asked to come for PET-CT with at least 4-h fasting. None of our patients were diabetic. The images were obtained on a dedicated PET-CT scanner (Siemens, Biograph-2), 60 min after intravenous injection of 370 MBq of ^18^F-FDG. CT acquisition was performed on spiral dual-slice CT with a slice thickness of 4 mm and a pitch of 1. Image was acquired using a matrix of 512×512 pixels and a pixel size of 1 mm. After transmission scan, three-dimensional (3D) PET acquisition was done for 3 min per bed position for 5–6 bed positions. PET data were acquired using matrix of 128×128 pixels with a slice thickness of 1.5 mm. CT-based attenuation correction of the emission images was employed. PET images were reconstructed by iterative method ordered subset expectation maximization (2 iterations and 8 subsets) with a filter size of 5 mm in this study. After completion of PET acquisition, the reconstructed attenuation corrected PET images, CT images, and fused images of matching pairs of PET and CT images were available for review in axial, coronal, and sagittal planes and in maximum intensity projections, 3D cine mode.

### Image analysis

Attenuation corrected and uncorrected images were displayed on the monitor. Both the attenuation corrected and uncorrected images were visually analyzed for abnormal FDG uptake by an observer who had more than 3 years experience of reading PET-CT. After image reconstruction, a region of interest (ROI) consisting of 9×9 mm was carefully drawn on the consecutive 4–6 PET slices. From these ROIs, SUVmax was calculated according to the formula described below:

$$\mathrm{Mean}\;\mathrm{ROI}\;\mathrm{activity}\;\left(\mathrm{MBq}/g\right)\times \mathrm{body}\;\mathrm{weight}\;\left(g\right)/\mathrm{injected}\;\mathrm{dose}\;\left(\mathrm{MBq}/g\right)$$

(MBq = a mega-Becquerel, and g = grams).

For determination of lymph node size, maximum diameter in one plane was used on CT images. In case of multiple lymph node involvement, largest node was considered for size evaluation.

### Histopathologic diagnosis

The histopathologic diagnosis was established in all the patients by any one of the following methods: peripheral lymph node biopsy, image-guided, mediastinoscopic, transbronchial, or mediastinal lymph node biopsy.

### Statistical analysis

The histopathologic diagnosis was considered the gold standard. The PET-CT data were expressed as mean (SS) and or median (range) as applicable. Qualitative and quantitative data were compared by Chi-square test, Student’s *t* test, Mann–Whitney *U* test, or analysis of variance as appropriate. A *P* value of <0.05 was considered significant. The sensitivity, specificity, PPV, NPV, and accuracy of PET-CT to differentiate benign and malignant lesions were calculated. A receiver-operator characteristic (ROC) curve was drawn to find out a cutoff value of SUV at which sensitivity and specificity are highest.

## Results

**Figure 1:**
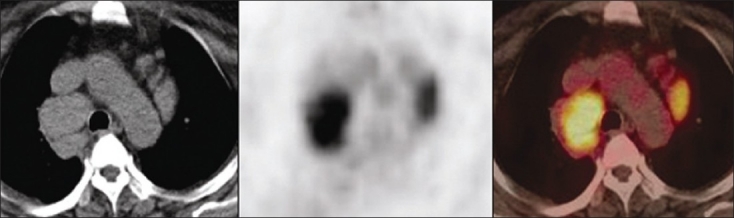
Axial CT, PET, and PET-CT images showing increased FDG uptake in mediastinal lymph nodes in a patient having tuberculosis

**Figure 2:**
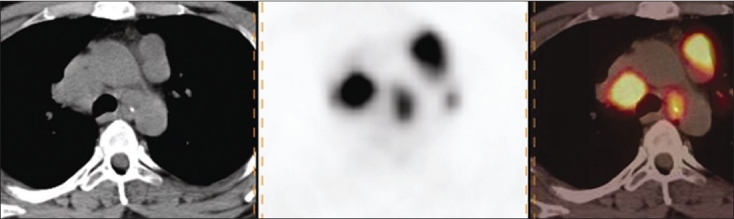
Axial CT, PET, and PET-CT images showing increased FDG uptake in mediastinal lymph nodes in a patient having sarcoidosis

**Figure 3:**
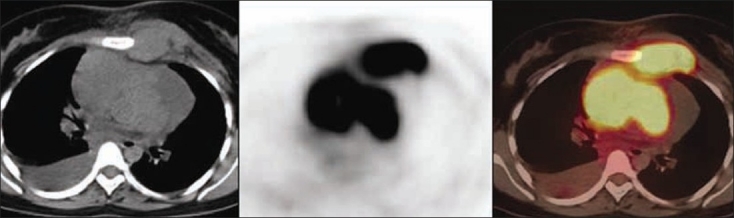
Axial CT, PET, and PET-CT images showing increased FDG uptake in mediastinal lymph nodes and left internal mammary lymph nodes in a patient having lymphoma

**Figure 4:**
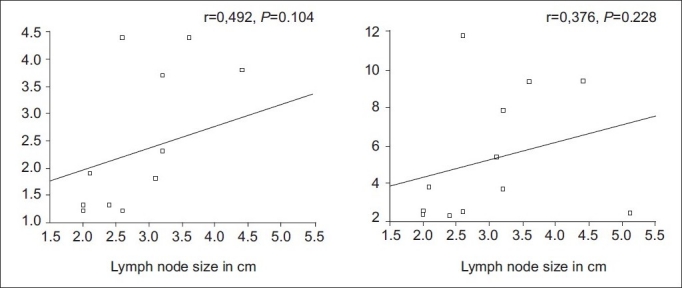
The maximum and average SUV correlation with the size of the mediastinal lymph nodes in tuberculosis

**Figure 5:**
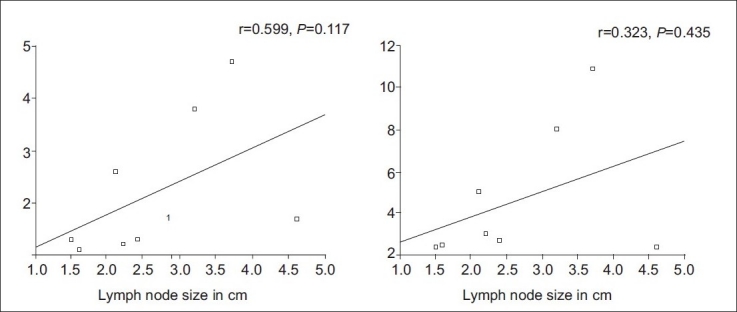
The maximum and average SUV correlation with the size of the mediastinal lymph nodes in sarcoidosis

Of the 35 patients, 20 were found to have benign, whereas 15 had malignant pathologies on histopathology. Among the 20 benign pathologies, 12 were tuberculosis [Figure 1] and 8 were sarcoidosis [Figure 2]. All the 15 patients in the malignant group had lymphoma [Figure 3]. The SUVmax of the benign nodes ranged from 2.3 to 11.8 with a mean±SD of 5.02±3.26. The SUVmax of the malignant nodes ranged from 2.4 to 34 with a mean±SD of 10.8±8.12. There was a statistically significant difference between benign and malignant pathology (*P*<0.0059) by Mann–Whitney *U* test. Similarly, average standardized uptake value (SUVavg) of the benign nodes ranged from 1.1 to 4.7 with the mean±SD of 2.3±1.28. The SUVavg of the malignant nodes ranged from 1.2 to 15 with the mean±SD of 4.59±4.24. There was a statistically significant difference between benign and malignant pathology (*P*<0.01) by Mann–Whitney *U* test. Table 1 shows the results of PET-CT scan with their histopathologic diagnosis. Among the benign pathologies, the SUVmax ranged from 2.4 to 10.9 with a mean±SD of 4.6±3.2 in sarcoidosis, while in tuberculosis the SUVmax ranged from 2.3 to 11.8 with a mean±SD of 5.3±3.4. The SUVavg in sarcoidosis ranged from 1.1 to 4.7 with a mean±SD of 2.2±1.36. The SUVavg in tuberculosis ranged from 1.2 to 4.4 with a mean±SD of 2.4±1.3. The difference in SUV max and average standardized uptake value (SUVavg) between 2 benign conditions was not statistically significant. The SUVmax and SUVavg were found to be correlating well with the size of benign mediastinal lymph node, but it was not statistically significant [Figures 4 and 5].

**Figure 6:**
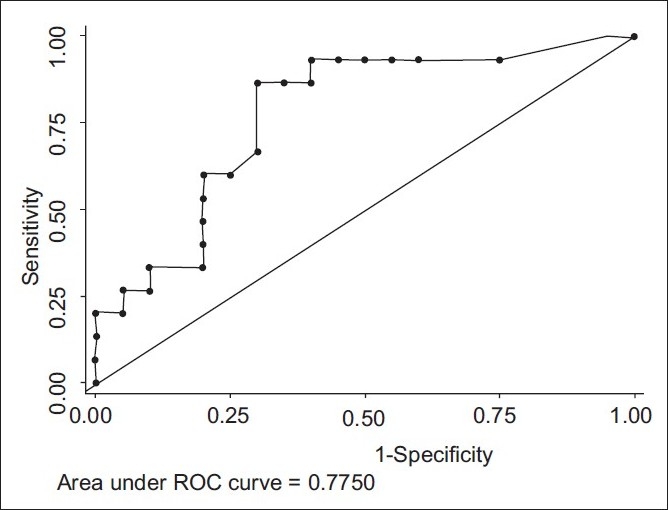
Receiver-operator characteristic curve to find out theoptimal SUVmax cutoff value

The SUVmax was used for discrimination between benign and malignant lymph nodes. We evaluated the efficacy of PET-CT at different cutoff values of SUVmax after reconstruction of a 4×4 table for each value. When 2.5 was used as the cutoff value, the sensitivity, specificity, PPV, NPV, and accuracy were 93%, 40%, 54%, 89%, and 63% respectively. With SUVmax of 5.3 as the cutoff as reported by Lee *et al*., for non-small cell lung cancer, the specificity in our study increased to 65% from 40% with no significant change in sensitivity, which decreased from 93% to 87%. In addition, accuracy also improved from 63% to 74%. We found the optimal SUVmax cutoff to be 6.2 as determined by the ROC curve analysis [Figure 6]. SUVmax of 6.2 as the cutoff value, the sensitivity, specificity, and accuracy were 87%, 70%, and 77%, respectively [Table 2].

**Table 2:** Efficacy of PET-CT at various cutoffs in differentiating benign from malignant mediastinal lymph nodes

SUVmax	Sensitivity (%)	Specificity (%)	PPV (%)	NPV (%)	Accuracy (%)
2.5	93	40	54	89	63
5.3	87	65	65	87	74
6.2	87	70	68	87	77

PET-CT, positron emission tomography-computed tomography; SUVmax, maximum standardized uptake value; PPV, positive predictive value; NPV, negative predictive value.

## Discussion

Mediastinal lymph node enlargements are caused most often by lymphoma, metastatic carcinoma, sarcoidosis, and infection, either directly by organisms, such as *Mycobacterium tuberculosis, Histoplasma capsulatum*, and others, or as a hyperplastic reaction to the presence of organisms within the lungs.[5–10] Noninvasive tests, such as CT and MRI have a limited role in differentiating malignant from benign lymph node due to lack of specific clinical parameters. Mediastinal lymph node staging has important therapeutic and prognostic implications in lung cancer. Patients without lymph node involvement are treated with surgery alone, whereas others with gross mediastinal lymphadenopathy are treated with definitive chemotherapy and radiotherapy.[11,12]

Distinction between benign and malignant involvement in isolated mediastinal lymph node requires invasive modalities, such as image-guided biopsy (CT, endoscopic ultrasound), transbronchial lymph node biopsy, or thoracoscopic or mediastinoscopic biopsy. Being invasive, each has its own list of complications. ^18^F-FDG PET-CT, a recently introduced noninvasive imaging modality, has been included in the staging of lymphoma and has been found to have higher sensitivity than CT.[13] However, FDG is not specific for detecting malignant conditions. It has been shown that inflammatory cells also show avidity for FDG.[14]

The SUVmax has been reported to be an independent predictor of malignancy and lymph node metastasis.[11] SUVmax is preferred over SUVavg as there is a variability of about 35% between observers when SUVavg is used and this reduces to 3% when SUVmax is used.[15] The SUVmax cutoff value of 2.5 is used commonly to differentiate between benign and malignant lesions.[16] A retrospective study from South Korea on focal abnormalities in pulmonary parenchyma showed that 90% of the tuberculoma showed SUVmax uptake above 2.5, thus supporting the need to increase the cutoff value to differentiate benign and malignant lesions.[17] We also observed similar results. There is a significant overlap in the SUVmax between benign and malignant conditions when SUV of 2.5 was used as the cutoff limit. In our study, there were 12 patients of benign condition with SUVmax more than 2.5. The sensitivity and specificity in our study was 93% and 40%, respectively, with 2.5 as the cutoff value. The low specificity observed with the cutoff of 2.5 could be due to high prevalence of inflammatory diseases in our patient population. Twenty of our cases (57%) were either tuberculosis or sarcoidosis.

In our study, highest SUVmax in patients of tuberculosis and sarcoidosis was 11.8 and 10.9, respectively. Similar higher values of SUVmax have been reported in the literature.[18] Lee *et al*. studied 110 patients of lung cancer to find out the utility of SUVmax in predicting malignancy in mediastinal lymph nodes using FDG PET-CT. Authors opined that SUVmax of 5.3 as cutoff increases the specificity from 86% to 98% and accuracy from 87% to 97%. In this study, the specificity increased from 40% to 65% when cutoff SUV was increased from 2.5 to 5.3.[11] However, in the present study, the best cutoff value with optimal sensitivity and specificity as determined by the ROC curve was 6.2. We found a sensitivity, specificity, and accuracy of 87%, 70%, and 77%, respectively, with SUVmax cutoff value of 6.2.

## Conclusion

The conventional SUVmax of 2.5 to differentiate benign and malignant lesions may not provide significant specificity in the developing countries where tuberculosis and sarcoidosis are common. Increasing the cutoff values can improve the specificity, while maintaining an acceptable sensitivity. A study incorporating large number of patients is needed to confirm our initial results.
